# Supplementary material for: Does Trypsin Oral Spray (Viruprotect®/ColdZyme®) Protect against COVID-19 and Common Colds or Induce Mutation? Caveats in Medical Device Regulations in the European Union
Source: Int J Environ Res Public Health. 2021 May 11;18(10):5066. doi: 10.3390/ijerph18105066 (PMC8150360; doi:10.3390/ijerph18105066)
Supplement: Supplementary file 1 [file ijerph-18-05066-s001.zip › ijerph-1172001-supplementary.pdf]

Table S1: Effects of trypsin exposure on infectivity/syncytia formation and mutations of spikes [18-36]

Virus	Results<br>+/- trypsin	Trypsin concentration	Author
	SARS-CoV		
SARS-CoV	Assays/methods:<br>• Baby Hamster Kidney (BHK) cells were treated with 1 μg/mL trypsin for 30 min or 1 h.<br>• VeroE6 (Vero C1008) cells were treated with 2 μg/mL trypsin for 20 min, then incubated for 40 min in absence of trypsin and analyzed by immunofluorescence microscopy using an anti-S monoclonal antibody, with nuclei counterstained with Hoechst 33548.<br>• BHK cells cotransfected with plasmids encoding the SARS-CoV S wild type (SARS wt) or SARS-R667N, as well as a plasmid encoding luciferase under the control of the T7 polymerase were overlaid with VeroE6 cells expressing the T7 polymerase. Cell–cell fusion was induced by treating the cells with increasing amount of trypsin for 20 min. At 5 h after fusion induction, the cells were lysed to monitor luciferase activity.<br>Results:<br>Infection of SARS-CoV could be strongly induced by trypsin treatment.<br>Arginine 667 was shown to be required for robust augmentation of SARS-S-driven cell-cell fusion by trypsin and for trypsin-dependent circumvention of entry blockade imposed by lysosomotropic agents. A proteolytic cleavage site within the SARS-CoV S2 domain (S2′, R797) is trypsin-activated.	1-3 μg/mL	Belouzard 2009 [18]
SARS-CoV	Assays/methods:<br>• Purified triSpikeprotein was first incubated with various proteases including trypsin, transmembrane serine protease (TMPRSS11a) and plasmin (0.2 mU of each proteases) in the presence of 0.1 MTris-HCl, pH 7.5 at 37°C for 2 h. Purified triSpike protein incubated without proteases was included as control. Samples were then subjected to sodium dodecyl sulfate polyacrylamide gel electrophoresis (SDS-PAGE) and visualized for cleavage products.	0.2 mU	Kam 2009 [19]

	• While pre-attachment, enforced trypsin and plasmin digestion (1 mg/mL) had no enhancing effects on virus entry in the stepwise host model, the effect of airway proteases on SARS-CoVpp entry after virus-cell attachment was studied in 16HBE cells pre-incubated with SARS-CoVpp or lentiviral vector without surface glycoprotein, hence allowing virus attachment but not virus entry. Unbound SARS-CoVpp or empp was washed away and proteases were added.<br>Results:<br>S was readily cleaved in vitro by three different airway proteases: trypsin, TMPRSS11a, plasmin. Trypsin recognized and cleaved peptides at both position R667 and R797 more efficiently than the other proteases. Sequence analysis of cleavage products showed that trypsin, plasmin and TMPRSS11a proteases cleaved S glycoprotein at positions 667 and 797. [Amino acid residue 667 has been proposed to be the potential cleavage site in Spike generating the S1 and S2 subunits.]<br>Under these conditions airway protease treatment with trypsin significantly enhanced the infection by triSpike pseudotypes. Also various concentrations of proteases were tested. The enhancement of SARS-CoVpp entry increased with higher concentration of the proteases.	 <br><br><br><br>0.1-10 μg/mL	
SARS-CoV	Assays/methods (Virus–virus fusion assay):<br>• Equal amounts of pseudovirions bearing either angiotensin-converting enzyme (ACE2) and encoding luciferase as a reporter Human Immunodeficiency Virus ((HIV-luc(ACE2)) or both SARS-CoV Spike (or mutants) and Avian Sarcoma Leukosis Virus-A envelope and encoding Green Fluorescent Protein (GFP, HIV-gfp(S + E)) were mixed and incubated for 30 min on ice to allow binding. The temperature was then raised to 37 °C for 15 min to allow induction of conformational rearrangements. Particles were then either treated with 10 ug/ml Thermo Scientific Pierce (TPCK)-trypsin or the pH was lowered to pH 6 by the addition of 0.1 M citric acid, and pre-activated recombinant cathepsin L was added to a final concentration of 2 ug/ml. Proteolysis was halted after 10 min at 25 °C by addition of soybean trypsin inhibitor and leupeptin. Virus mixtures were then diluted and used to infect HeLa cells.<br>Results:<br>Trypsin treatment was required for cell-cell fusion of SARS-S.	10-15 μg/mL<br>10 min<br>at 37°C	Simmons 2011 [20]

	Residue R667 but not K672 determines sensitivity of SARS-S to inactivation by trypsin itself.<br>Cell-free virions bearing WT SARS but not mutant R667A were activated by trypsin.<br>[The effect of introduction of residues on cell-cell and virus-cell fusion typical of mutants is described, leading to augmented infectivity: e.g. mutation T760R optimizes the minimal furin consensus motif 758-RXXR-762; and furin overexpression augmented SARS-S activity.]		
	SARS-CoV-2		
SARS-CoV-2	Assays/methods:<br>• Nasopharyngeal aspirate specimen were incubated on VeroE6 cells, with and without 0·5 μg/mL trypsin. Inoculated cells were monitored daily for cytopathic effects by light microscopy, and cell supernatant was collected daily for quantitative reverse transcription polymerase chain reaction (RT-PCR) to assess the viral load.<br>• Assay of viral replication in various human (n=9) and non-human host cell lines (n=16) +/- trypsin.<br>Results:<br>Substantial cytopathic effects were seen at 72 h post inoculation. Trypsin was not reported to stop infectivity of SARS-CoV-2 in any of these 25 in vitro models.	0·5 μg/mL	Chu 2020 [21]
SARS-CoV-2	Assays/methods:<br>• For spike activation by trypsin treatment, viral pseudoparticles were treated with 2.5mg/mL trypsin for 1 h at 37˚C before addition to target cells overexpressing human ACE2. Among others, SARS-CoV-2pps, SARS-CoVpps, along with their respective negative controls were titrated 10-fold on target cells and incubated for 72h at 37℃, 5% CO2.<br>• For cell-cell fusion assay, protease activation was tested in 3 different conditions, one of which using effector cells that were washed twice with phosphate-buffered saline (PBS) and incubated with 3μg/mL of TPCK-treated trypsin for 30 min at 37℃.<br>Results:	2.5mg/mL<br>3 μg/mL	Conceicao 2020 [22]

	Co-expression of human ACE2 and TMPRSS2 in target cells or separate trypsin exposure of S expressing effector cells both led to larger syncytia when compared with ACE2 alone.<br>[Attributed to the result of direct activation of a greater % S proteins at the cell–cell interface]		
SARS-CoV-2	Assays/methods:<br>• For the syncytium formation assay, Vero or Vero-TMPRSS2 cells were transfected to express the indicated S proteins (or no S protein, empty vector, control). At 24 h post transfection, cells were incubated in the presence or absence of trypsin (1μg/mL) for an additional 24 h before they were fixed for staining and analysis by bright field microscopy.<br>Results:<br>SARS-2-S expression triggered syncytium formation that was strongly increased by trypsin.	1 μg/mL	Hoffmann 2020 [23]
SARS-CoV-2	Assays/Methods:<br>• Exogenous trypsin was used to mimic TMPRSS2 at the cell surface as the two enzymes are closely related and both belong to the group of trypsin-like proteases. Viral particles were first subjected to proteases prior to being added to Caco-2 and Vero cells.<br>• In the cell-cell fusion assay, large syncytia with five or more nuclei were observed when infected Vero cells were exposed to trypsin.<br>Results:<br>Infection increased as much as 2- to 3-fold following the SARS-CoV-2 proteolytic processing by trypsin, whereas the pre-exposure of particles to furin had no apparent effect.<br>Proteolytic processing of S with trypsin triggers SARS-CoV-2 membrane fusion (well-illustrated in Figure 6). SARS-CoV-2 after proteolytic processing. Once activated by trypsin proteolytic cleavage, the virus was no longer required endosomal acidification for infection.	100 μg/mL<br>5 min<br>at 37°C	Koch 2020 [24]
SARS-CoV-2	Assays/methods:<br>• Cell–cell fusion assay: Human Embryonic Kidney (HEK)-293T cells were co-transfected with plasmids encoding CoV S glycoprotein and eGFP. For trypsin-dependent cell–cell fusion, cells were detached with trypsin (0.25%) at 40 h post transfection and overlaid on an 80% confluent	0.25%	Ou et al. 2020 [25]

	monolayer of 293/hACE2 cells at a ratio of approximately one S-expressing cell to three receptor-expressing cells. For trypsin-independent cell–cell fusion, cells were detached with 1 mM ethylene-diaminetetra-acetic-acid (EDTA) and overlaid on 293/hACE2 cells. After 4 h incubation, images of syncytia were captured with epifluorescence microscopy.<br>• Results:<br>Addition of trypsin triggered syncytium formation of 293/hACE2 cells by S protein after 4 h of incubation.<br>[0.25% also caused cell detachment]		
SARS-CoV-2	Assays/methods:<br>• Material from the initial nasopharyngeal swab was used to inoculate a Vero/hSLAM cell line. Flasks were monitored for the development of viral cytopathic effect and 140 µL aliquots removed every 48 hours to assess virus burden by real time RT-PCR.<br>• For electron microscopy, a 4 mL aliquot of supernatant from cell cultures grown in the presence of 4 µg/mL trypsin was inactivated with 0.5% glutaraldehyde for 12 h and clarified by centrifugation at 1000 g for 3 min<br>Results<br>Isolation of SARS-CoV-2 from the first patient diagnosed with COVID-19 in Australia by growing the isolate in cell cultures in the presence of trypsin. Following several failures to recover virions with the characteristic fringe of surface spike proteins, it was found that adding trypsin to the cell culture medium immediately improved virion morphology.	4 µg/mL	Caly 2020 [26]
SARS-CoV-2	Assays/methods:<br>• Fusion rates of cell–cell fusion between target cells and series of effector cells in the presence of indicated concentration of trypsin (h) or HAT(i), taking the fusion rate of SARS-CoV-2 group treated by exogenous trypsin (200 ng/ml) or HAT(500 ng/ml) as 100%. …/…<br>Results:<br>Fusion rate is enhanced the higher the concentration of trypsin. **Mutants** without functional furin-like cleavage (FLC) site and SARS-CoV-S also effectively mediate cell–cell fusion in a trypsin	50-200 ng/mL	Xia 2020 [27]

	concentration-dependent manner; high concentration of trypsin could entirely recover the fusogenic capacity of SARS-CoV-2-S without FCS.		
SARS-CoV-2	Assays/methods:<br>• To assess whether exogenously added trypsin could compensate for TMPRSS2 deficiency and the emergence of S gene mutants during SARS-CoV-2 propagation, Vero,Vero-TMPRSS2, Calu-3, Caco-2, and293T ACE2cells in complete culture medium or (forVero) in serum free Dulbecco's Modified Eagle's Medium (DMEM) supplemented with 0.5µg/ml trypsin were used. Virus propagation was performed in 12-well plates; culture supernatant was collected at day 3 post-infection and used to inoculate naïve cells for virus passage. Ribonucleic acid (RNA) was extracted from the culture supernatant after each passage.<br>Results:<br>S gene **<u>mutants</u>** did emerge and accounted for the majority of the virus population after 2 passages in Vero cells cultured in serum-free medium with added trypsin. S gene mutants have been commonly reported on passage in Vero cells but they were not detected in this study during the propagation in TMPRSS2 expressing cells such as Vero-TMPRSS2 (without trypsin).	0.5 µg/mL	Sasaki 2020 [28]
SARS-CoV-2	Assays/methods:<br>• Particles were allowed to bind for 1 h at 4°C after which unbound particles were removed and DMEM as such, with 10 µg/mL TPCK-trypsin, was added. After 2 h at 37°C, the medium was changed again to complete medium with 10% FCS. Each trypsin-treated condition was compared to untreated…/…<br>Results:<br>Three SARS-2-SD614 **<u>mutants</u>** bearing deletions in the S1/S2 cleavage loop showed markedly reduced entry into Calu-3 cells (6-to 30-fold; P ≤ 0.009 versus Wild Type). Entry was fully rescued by exogenous trypsin if added during Calu-3 cell transduction, indicating that the poor entry was due to a lack of S2' cleavage and not to inefficient receptor binding.	10 µg/mL	Laporte 2020 [29]

Lineage B betacorona-viruses, including SARS-CoV and SARS-CoV-2	Assays/methods:<br>• African green monkey (AGM), human or bat cells were infected with vesicular stomatitis virus (VSV) particles pseudotyped with lineage B chimeric spikes. Pseudotypes were either left untreated or incubated with trypsin before addition to the cells. Luciferase was measured and normalized to particles produced without spike.<br>• Pseudotypes were left untreated or treated with trypsin and subsequently used to infect Huh-7.5 cells.<br>• Pseudotypes with the indicated spike constructs were left untreated or treated with trypsin and subsequently used to infect BHK cells expressing human ACE2.<br>• At last, pseudotyped particles were left untreated or treated with trypsin and subsequently used to infect BHK cells expressing the indicated coronavirus receptors.<br>Results:<br>Host protease processing during viral entry is a significant barrier for several lineage B viruses. Trypsin enhanced lineage B viral entry in various cell lines. Further studies are needed to assess where trypsin is enhancing entry of coronaviruses at the level of S, receptor, or both.	-	Letko 2020 [30]
Other respiratory coronaviruses			
Avian Coronavirus IBV (gamma - coronavirus)	Assays/methods:<br>• Infection of Vero and DF-1 Cells with infectious bronchitis virus (IBV) in the presence of TPCK-Treated Trypsin: Six-well tissue culture plates of Vero or DF-1 cells were infected with IBV at a multiplicity of infection (MOI) of 0.1, diluted in serum free N,N-bis(2-hydroxyethyl)-2aminoethanesulphonic acid (BES) medium with added trypsin at concentrations of either 0.2, 1.0 or 2µg/mL for Vero or 0.2, 0.5 or 1.0µg/mL for DF-1 cells, in a total volume of 500µL per well. BES medium alone or BES medium containing the same concentrations of trypsin as infected wells was used for mock infections. Cells were incubated at 37◦C in 5% $CO2$ for 1 h.	0.2, 1.0 or 2µg/mL for Vero<br><br>0.2, 0.5 or 1.0µg/mL for DF-1 cells	Stevenson-Leggett 2020 [31]

	• Following the primary incubation step, the virus inoculum was removed, and cells were washed twice in PBS before the addition of 3 mL of BES medium with the appropriate concentration of trypsin (matched to the media in which the virus was diluted). Cells were incubated at 37◦C in 5% CO2 for 24h, after which the supernatant was harvested. Quantities of infectious viral progeny in the supernatant were assessed by titration in CK cells.<br>• To prepare Vero cell lysates, cells were washed once in cold PBS after which 350μL of radio-immunoprecipitation assay (RIPA) lysis buffer. Cells were incubated on ice for 20 min with constant agitation. Cells were scraped into buffer and centrifuged at 10,000×g; supernatant was stored at−20◦C.2.3.<br>• Analysis of IBV Replication Kinetics in the Presence of TPCK-Treated Trypsin: confluent six-well tissue culture plates of Vero cells were infected with IBV M41-CK or recombinant IBV Beau-R at a multiplicity of infection (MOI) of 0.01. Viruses were diluted in serum-free BES medium containing TPCK-treated trypsin at a concentration of 1.0μg/mL or BES medium alone (untreated) in a total volume of 500μL<br>Results:<br>Addition of exogenous trypsin during IBV propagation in cell culture resulted in significantly increased viral titres and overcomes the barrier to infection upon serial passages in cell cultures. **Mutations** were identified in both S1 and S2 subunits.		
MERS pseudovirions	Assays/methods:<br>• Middle East Respiratory virus (MERS) pseudovirions with uncleaved S protein were adsorbed at 4°C to cell surface receptors on Vero E6 cells in the presence of 20mM ammoniumchloride, and then the cells with bound virions were briefly treated with trypsin at pH 7.4 at room temperature to cleave the ~200 kDa S protein and activate its membrane fusing activity.<br>Results:<br>Brief trypsin treatment at neutral pH triggered virus entry at the plasma membrane at neutral pH and caused massive syncytia formation even in cells that express little or no MERS-CoV receptor.	15 μg/mL TPCK-trypsin	Qian 2013 [32]
aMPV	Assay:		Yun 2015 [33]

(subfamily Pneumovirinae of family Paramyxoviridae)	• Syncytium formation assay: monolayers of Vero cells in 6-well plates were transfected with 2 μg of each plasmid using Lipofectamine 2000. The transfected cells were incubated with or without 0.2 μg/mL TPCK-trypsin for 24 h. After transfection, the cells were exposed to PBS at pH 5.0 (low pH) or pH 7.4 (neutral pH) for 4 min. This pH pulse was repeated three times at 2-h intervals. At 48 h post-transfection, the cells were observed and photographed.<br>• Reporter gene assay: to evaluate the fusogenic activity of avian Metapneumonovius (aMPV) F proteins, Lipofectamine 2000 was used to transfect Vero cells in 6-cm dishes with 2 μg of wild-type or mutant aMPV-F and 1 μg T7 control plasmid containing luciferase copy deoxyribonucleic acid (cDNA, under control of T7 promoter). The transfected Vero cells were incubated with or without 0.2 μg/mL of trypsin for up to 24 h. The transfected Vero cells and BHK-21 cells infected with the modified vaccinia Ankara (MVA-T7) at a MOI of 1 were treated with PBS (pH 5.0 or pH 7.4) after transfection. The next day, the Vero cells were lifted, resuspended and overlaid onto infected BHK-21 cells. At 48 h post-transfection, the cell lysates were analysed for luciferase activity<br>Results:<br>Trypsin is required for the in vitro propagation of hMPV strains.<br>Trypsin boosted the fusogenic activity of aMPV F 100Q101S **<u>mutants</u>** of all examined aMPV subtypes.	0.2 μg/mL<br><br>0.2 μg/mL	
Middle East respiratory syndrome (MERS)-like bat CoVs	Assay:<br>• Replication in Vero, Calu-3 2B4, Caco-2, Huh7, and human airway epithelial (HAE) cells was assessed: cells were washed with PBS and inoculated with virus or mock diluted in Opti-MEM (minimal essential media) for 60 min at 37°C. Following inoculation, cells were washed three times. Fresh medium with or without trypsin was added to signify time zero. For HAE cultures, apical surfaces were washed with PBS containing 5μg/mL trypsin at 0, 8, 18, 24, and 48 hours post-infection (microscopic analysis).<br>• Virus neutralization assays. Plaque reduction neutralization titer assays were performed with antibodies against MERS-CoV. These were serially diluted 6- to 8-fold and incubated with 80 plaque-forming units (PFU) of the indicated viruses for 1h at 37°C. The virus and antibodies	0.5μg/mL	Menachery 2020 []34

	were then added to a 6-well plate of confluent Vero cells in triplicate. After 1 hour incubation at 37°C, cells were overlaid with 3 ml of 0.8% agarose in Opti-MEM supplemented with 0.5 μg/mL trypsin.<br>Results:<br>The S protein of PDF2180-CoV, a MERS-like virus found in a Ugandan bat, mediated infection of Vero and human cells in the presence of exogenous trypsin. Addition of exogenous trypsin also rescues HKU5-CoV, a second bat group 2c CoV.		
Other viruses			
Porcine epidemic diarrhea virus (α-coronavirus)	Assays/methods:<br>• Virus isolation and propagation: porcine epidemic diarrhea virus (PEDV) was isolated from Vero cells. The supernatant of intestinal homogenates or fecal samples was filtered. Confluent Vero cells were then washed three times with DMEM, and inoculated with 1 mL of the above supernatant and 4 mL of post-inoculation medium by adding trypsin (Gibco, USA) to a final concentration of 2.5, 10, 20, 30 ng/μL, respectively. The cells were incubated at 37 °C with 5% CO2 for 3 days.<br>• Virus titration and growth characteristics : washed confluent Vero cells were infected with the parent 85-7 strain (Passage 5, P5) or the isolated mutants at a multiplicity of infection (MOI) of 0.1 in the presence (10 ng/μL) or absence of trypsin at 37 °C for 1 h, after which the incubation virus was discarded. The infected cells were washed three times with DMEM, cultured at 37 °C by adding the maintenance medium (DMEM supplied with 2% FBS), and the culture supernatants were collected at different time points [12, 24, 36, 48, and 60 h post infection (hpi)]. As to the trypsin-dependency assay, Vero cells were infected with parent 85-7 strain at an MOI of 0.1 within different concentrations trypsin or absence of trypsin, and the culture supernatants were collected at 48 hpi. Finally, titers of the culture supernatants at the indicated times were determined by plaque assay on Vero cells, and quantified pfu per milliliter (mL).	10 ng/mL	Sun 2017 [35]

	• For the viral binding assay, cells were incubated with the same amount of parent 85-7 (P5), C30 or C40 viruses at 4 °C for 1 h in the presence (10 ng/μL) or absence of trypsin. After that the samples were washed three times with DMEM, then the binding efficiency was determined by plaque assay. The entry assay was performed following the viral attachment step. The samples were maintained in DMEM at 37 °C for 1 h in the presence (10 ng/μL) or absence of trypsin, and the cells were washed with cold acidic PBS (pH 3.0) to remove the virus binding to the cell membrane, then the entry efficiency was determined by plaque assay.<br>Results:<br>Propagation of PEDV isolates requires supplementation with trypsin in the cell culture supernatant in vitro. **Mutation**s in its S and envelope proteins allow to proliferate in absence of trypsin.		
Swine acute diarrhea syndrome coronavirus (recombinant SADS-CoV)	Assays/methods:<br>• One-step growth curves were used to assess virus growth in swine LLC-PK1 and primate Vero CCL-81 kidney cultures. All supernatants were titered on Vero CCL-81 cells. Cultures of LLC-PK1 cells infected with SADS-CoV and protein lysates were run on Western blots with and without trypsin for comparison. Cell lysates were probed with antinucleocapsid antibodies from sera of mice immunized with VRP. N proteins (41.6 kDa) were present among both conditions.<br>Results:<br>Efficient virus growth was shown in swine LLC-PK1 and primate Vero CCL-81 kidney cultures in the presence, but not absence, of low levels of trypsin	10 μg/mL	Edwards 2020 [36]
